# Crosstalk between glomalin and AMF reduces cadmium uptake and leaching, enhancing pea yield and soil health

**DOI:** 10.1186/s12870-025-07534-2

**Published:** 2025-11-07

**Authors:** Muhammad Usama, Muhammad Shoaib Khan, Fakhir Hannan, Bilal Rasool, Hina Rizvi, Muniba Farhad, Hafiz Muhammad Tauqeer, Sami Ullah, Muhammad Iqbal

**Affiliations:** 1 Department of Environmental Sciences, Government College University Faisalabad, Faisalabad, 38000 Pakistan; 2 Department of Primary Industries and Regional Development, 9 Modal Cres, Canning Vale, WA 6155 Australia; 3 College of Agriculture and Biotechnology, Zhejiang University, Hangzhou, 310058 China; 4 Department of Zoology, Faculty of Life Sciences, Government College University Faisalabad, Faisalabad, 38000 Pakistan; 5 Department of Chemistry, Government College University Faisalabad, Faisalabad, 38000 Pakistan; 6 Department of Environmental Sciences, University of Gujrat, Gujrat, 50700 Pakistan; 7 Department of Chemistry, College of Science, King Khalid University, Abha, 61413 Saudi Arabia; 8 Research Centre for Advanced Materials Science, King Khalid University, Abha, 61413 Saudi Arabia

**Keywords:** Untreated effluents, Pottery, Human health, Synergism, Glomalin, Permissible limits

## Abstract

**Background:**

Untreated effluents from the pottery industry pose ecological and human health risks by polluting soil, food crops, and groundwater with cadmium (Cd). Arbuscular mycorrhizal fungi (AMF) and easily extractable glomalin (EG) secreted by AMF can immobilize Cd in soil and minimize its migration to food crops and groundwater. This study hypothesized that applying EG and AMF together in Cd-polluted soil may result in their synergistic crosstalk. This synergism can reduce Cd migration from soil to plants and groundwater, and improve plant traits and soil health compared to the sole application of AMF and EG. This pot study investigated a novel idea: amending pottery Cd-polluted soil with sole AMF inoculum, EG, and AMF + EG. Later, Cd bioavailability in soil and its migration in pea plants and leachates, as well as plant growth and yield, grain nutrition, and activities of soil enzymes, were examined.

**Results:**

A synergistic interaction between AMF and EG occurred in the AMF + EG treatment, which reduced the concentrations of bioavailable Cd in soil, plant shoots, roots, and grain by 64%, 68%, 53%, and 89%, respectively, while improving grain nutrients than untreated control treatment (CK). Surprisingly, Cd concentrations in grain and leachates were below the permissible limits of FAO and WHO for food and drinking water safety with AMF + EG. Moreover, maximum improvements in dry weight of pea shoot (36%) and root (60%), pod numbers (144%), and grain yield (58%) were noted with AMF + EG than CK. The highest rise in soil enzymatic activities, i.e., β-glucosidase (68%), urease (72%), catalase (206%), chitinase (87%), and phosphomonoesterase (172%) with AMF + EG compared to CK, indicated maximum improvements in soil health.

**Conclusions:**

Synergism between AMF and EG in AMF + EG treatment can efficiently remediate Cd-polluted soils, reduce Cd migration to food and groundwater, and improve soil and plant health.

## Introduction

Ceramic products like glazed interior and wall-floor tiles, sanitaryware, tableware, and ceramic mosaic are manufactured through advanced ceramic processes [1, 2], via firing at higher temperatures (900–1500 °C) [3]. Lead (Pb) is used as a fluxing agent, which reduces firing temperature, whereas Pb compounds, like lead silicate (Pb-Si-O) and lead oxide (PbO), are used as a glaze for ceramic products [4, 5]. The ceramic pigments are metal transition oxides [2], which retain color, tinting strength, and chemical stability at high firing temperatures [6]. To color the glazed ceramic materials, chromium (Cr) pigments are preferred due to their chemical resistance. While the cadmium (Cd) ones are not a good option due to their poor thermal stability, oxidation, and color loss at higher temperatures [7, 8].

Whereas, crude pottery (earthenware) is a significant part of unglazed handicraft products made from clay fired at a lower temperature [9, 10]. These products exhibit several features, including weak frost resistance, a porous structure, low strength, a rough and non-luminous appearance, and require low sintering, fracture surface, and harsh knocking [2]. These products are sun-dried, fired red in a kiln, and decorated with different geometrical, floral, animal, anthropomorphic, and structural patterns [9]. Earthenware products are decorated with brilliant yellow, orange, red, and rainbow colors developed from various Cd pigments (Cd selenide (CdSe), Cd sulfide (CdS), and Cd sulfoselenide (Cd_2_SSe)) at low temperatures [11]. These pigments enhance their color permanence, thermal stability, chemical resistance, and brilliance [12]. The annual global consumption of Cd pigments exceeds 2,500 tons, and 9% of it is used by the ceramics industry [12].

Unfortunately, untreated effluents from the pottery industries may pollute the surrounding soil and groundwater with Cd [13]. In Castellon, Spain, applying ceramics biosolids (Cd ≤ 21 mg kg^‒1^) in five agricultural soils raised their Cd concentrations above Spanish regulations [14]. Likewise, the dustfall from ceramic industries in Yixing City, China, polluted city soil with Cd (≤ 19.4 mg kg^‒1^) and suburb soil (≤ 43.9 mg kg^‒1^). Cd pollution levels in atmospheric dustfall in these areas showed higher values of ecological hazard index (*E*_*i*_ = 219.1), geoaccumulation index (*I*_*geo*_ = 5.3), and pollution level (6), which may directly harm human health, especially children, more than adults after Cd exposure through dermal, oral, and inhalation pathways [15]. Unfortunately, a ceramic company in Ajaokuta, Nigeria, resulted in Cd buildup in surrounding agricultural soils above critical values [16]. Whereas workers in the ceramic industry of Birjand, Iran, exhibited elevated levels of Cd (8.90 ± 2.80 µg L^‒1^) in their blood, which caused Cd-induced cytotoxicity. This Cd-induced cytotoxicity resulted in oxidative stress, as indicated by a higher malondialdehyde (MDA) content and reductions in the activities of superoxide dismutase (SOD) and glutathione peroxidase (GPx) in their plasma [17].

Since Cd is highly soluble in farmlands, it migrates through rainfall and runoff towards surrounding ecosystems and groundwater via leaching, affecting their quality [18, 19]. Moreover, photo-dissociation of Cd from Cd_2_SSe polluted river water [12], while enhanced uptake of Cd in rice grain due to Cd dissolution from yellow pigments in plant rhizosphere was reported [20]. A higher Cd concentration in soil negatively affects plant growth by causing cellular injury, reducing the uptake of water and nutrients, and influencing their photosynthesis and metabolism [11, 21]. Consuming Cd-contaminated food causes dysfunction of the testicular, renal, liver, and neurological systems, DNA damage, skeletal ailments, and cancer in humans [11].

Mycoremediation, i.e., using fungi to decontaminate Cd-polluted soils, is an innovative, efficient, cost-effective, and eco-friendly approach [21, 22]. Features like Cd tolerance, vast hyphal network, vigorous growth, versatile cellular enzymes, and higher surface area to volume ratio make them suitable candidates for this purpose [22]. Among various fungi, arbuscular mycorrhizal fungi (AMF) are known to establish the oldest and most abundant plant–microbe symbiosis on earth [23], which can efficiently reduce Cd bioavailability in soil and its uptake in plants [24]. Herewith, AMF secrete glomalin and extracellular polymeric substances (EPS), which chelate Cd in soil. Moreover, physical obstruction to Cd entry into roots, mycelial sequestration, and enhanced phytochelatins (PCs) synthesis in roots are other AMF-linked mechanisms [19, 25]. Additionally, AMF boost plant growth by facilitating water and nutrient uptake and improving soil health [23, 26, 27].

AMF produce a glycoprotein named “glomalin” with low solubility, excellent stability, and a 50-year turnover time [23, 28]. Glomalin is divided into easily extractable glomalin (EG) and difficultly extractable glomalin (DG) fractions [29, 30]. EG is newly synthesized, highly reactive, resilient (≥ 200 °C), non-bound, sequesters C, and stabilizes soil structures. Meanwhile, DG is an old fraction originating from EG turnover and is soil-bound [30, 31]. Interestingly, EG firmly binds Cd through electrostatic interaction, complexation, and adsorption on active sites, various functional groups, and legends. Such EG features depict its potential to mitigate environmental and human health risks by reducing Cd mobility in soil-plant systems [32, 33]. Previously, EG addition in soil improved agronomic parameters and leaf nutrients of lemon seedlings [29]. Enhanced orange fruit quality and nutrition were achieved with EG spray [34].

As per previous findings, information about the interaction between AMF and EG remains significantly less known. Previously, higher AMF root colonization raised the production of EG and total glomalin (TG) in soil [35], representing a positive correlation between the percentage of AMF root colonization and soil contents of EG and TG [36]. Whereas, glomalin also positively boosted the soil AMF activities and root colonization through (1) improving soil organic carbon (SOC) status [37], and (2) providing nutrients [38].

To the best of our knowledge, this is the first attempt to test synergistic crosstalk between AMF and EG, which may provide valuable insights into strengthening the mitigation of ecological and human health hazards associated with Cd. We hypothesized that synergistic crosstalk between AMF and EG may exist, supporting each other’s functions and potentially reducing Cd mobility in the soil-plant system, thereby enhancing soil health and improving plant traits. Therefore, a pot study was conducted in which Cd-polluted pottery soil was amended with a sole AMF inoculum and EG, as well as AMF + EG. The aim of this study was to (i) explore the synergistic crosstalk between AMF and EG in terms of benefitting each other by investigating several AMF parameters and production of EG in the soil; (ii) effect of this crosstalk on Cd immobilization in Cd-polluted soil and its transference in pea plants and leachates; (iii) beneficial influences on pea grain safety, plant productivity, and grain nutrition, and (iv) activities of soil enzymes indicating soil health.

## Materials and methods

### Cd-polluted pottery soil

Cd-polluted soil (0–15 cm [top layer]) was collected from arable fields that frequently receive untreated effluents from different pottery industrial units for many years. These units make unglazed crude pottery products in the industrial estate of Gujrat, Pakistan. The soil was collected from these agricultural fields after verbal consent from the landlord. Furthermore, neither ethics approval nor official permits were required to collect soil samples from this location. The soil was initially air-dried and passed through a 2-mm sieve. Physicochemical properties of this soil were: texture = sandy loam, sand = 53.4%, silt = 27.2%, clay = 19.4%, organic matter (OM) = 0.41%, calcium carbonate (CaCO_3_) = 3.8%, electrical conductivity (EC) = 3.17 dSm^−1^, pH = 6.87, cation exchange capacity (CEC) = 11.2 cmol kg^−1^, available P = 6.19 mg kg^−1^, exchangeable K = 108.1 mg kg^−1^, Ca = 1.29 g kg^−1^, Mg = 41.3 g kg^−1^, total Cd = 5.87 mg kg^−1^, diethylenetriaminepentaacetic acid (DTPA)-extracted Cd = 0.64 mg kg^−1^, total Pb = 9.2 mg kg^−1^, DTPA-extracted Pb = 0.27 mg kg^−1^, total Cr = 2.16 mg kg^−1^, and DTPA-extracted Cr = 0.03 mg kg^−1^. The permissible limits of Pb, Cd, and Cr for the soil are 300, 3, and 150 mg kg^−1^ soil, respectively [39]. In this collected soil, the Cd concentration exceeded the permissible limit, while the Pb and Cr concentrations were far below their respective limits, indicating that this soil was only polluted with Cd.

### Soil amendments

#### Arbuscular mycorrhizal fungi

An AMF inoculum named “Micronized endomycorrhizal inoculant” containing nine species, i.e., *Glomus aggregatum*, *Glomus etunicatum*, *Glomus clarum*,* Glomus intraradices*, *Glomus monosporus*, *Gigaspora margarita*,* Paraglomus brasilianum*,* Glomus deserticola*,* and Glomus mosseae*, was used for soil inoculation. This inoculum had a minimum count of 50,000 AMF spores/propagules kg^−1^. This commercially available AMF inoculum was purchased from BioOrganics, Pennsylvania, USA (www.bio-organics.com) by one of our research colleagues during his stay in the USA and brought to Pakistan.

#### Easily extractable glomalin

EG was extracted from a mixture of glomalin-free coarse sand and coal [40]. This mixture was filled into plastic pots (15 cm diameter, volume = 1300 cc). Five corn seeds (*Zea mays*) and AMF inoculum (same as used for soil application) were placed in nylon mesh bags (diameter = 8 cm, mesh size = 38 μm). These bags were placed in the centre of each pot to retain plant roots and AMF hyphae, while a root-free hyphal chamber was situated around the nylon bags [41]. After 84 days of plant growth, the plants and nylon mesh bags with roots were discarded. The sand remaining in the pots containing hyphae was extracted with citrate buffer (pH = 7.0, 20 mM, sand to buffer ratio = 1:8, w/v), autoclaved (30 min, 121 °C, 0.11 MPa), and centrifuged (10,000 × g, 5 min) [42]. The concentration of EG in the citrate buffer was determined using the Bradford assay with bovine serum albumin as the standard [43]. The amount of EG extracted from the sand-coal mixture was 1.32 mg g^−1^. EG was precipitated, dialyzed against water, and freeze-dried [44]. During EG extraction, quality control was ensured by using sterile items, including pots, corn seeds, irrigation water, plastic vats, and nylon mesh bags. Moreover, each laboratory ware used during EG extraction was sterile. Analytical grade chemicals were used, and each instrument was precise and calibrated during EG extraction. The citrate buffer was freshly prepared, ensuring its molarity and pH. The conditions during autoclaving were ensured. Blank samples (deionized water) and reference material (bovine serum albumin) [43] were used to ensure the quality of EG on a spectrophotometer. The properties of EG were: color = light brown, C = 38.9%, H = 4.19%, O = 32.7%, N = 3.16%, P = 0.17%, K = 0.51%, Ca = 0.57%, Mg = 0.25%, S = 0.53%, Cu = 0.23%, Mn = 0.18%, Zn = 0.14%, Fe = 1.97%, and Si = 0.11% and pH = 6.8.

### Plant experiment

Four soil treatments, designated as untreated control (CK), AMF, EG, and AMF + EG, were prepared by mixing EG and AMF into the soil. In AMF treatment, the manufacturer-recommended dose of AMF inoculum (3.40 g kg^−1^ soil) was added. To prepare the EG treatment, 50 mg of EG was mixed into each kg of soil. This dose of EG was the most cost-effective and efficient for immobilizing Cd in this soil, as calculated from a preliminary incubation experiment. In AMF + EG treatment, full doses of AMF and EG were mixed. Neither AMF inoculum nor EG was added to the CK treatment. The treated soils were put into airtight resealable plastic bags, maintained 65% water holding capacity (WHC) by adding deionized water, and placed for incubation in a dark room (42 days, 25 °C). Later, 3 kg of each amended soil with three replicates of every treatment were put into plastic pots (D = 17 cm and H = 13 cm) with drainage apertures. A plastic mesh (pore size = 1.5 mm × 1.5 mm) was placed inside each pot before filling the soil to allow the leachates to pass through and avoid soil loss. All pots were placed at the open experimental site of Government College University, Faisalabad, Pakistan, and randomized. This experimental area is designated to conduct all plant growth experiments, both by faculty and research students of this university. Drip trays were placed under each pot to collect the leachates. Pea seeds of the variety “Classic” were bought from the “Asian seed company”, Gujranwala, Pakistan. Five pea seeds were planted in each pot, from which only three healthy plants per pot were kept after ten days of growth. Pots were irrigated with sterile water. In each pot, fertilizer named “Osmocote^®^ Smart-Release”, purchased from “https://miraclegro.com/en-us/home#”, was applied at one time after one month of plant growth. The positions of pots were weekly relocated. Immediately before collecting the leachates, old drip trays were replaced with new ones to avoid Cd contamination. After irrigating the pots, the leachates were collected on 25, 50, 75, and 100 days of plant growth. The leachates were collected with sterile syringes, filtered with disposable polyethersulfone syringe filters, acidified (100 µL of 65% HNO_3_), and stored in glass vials (4 °C).

After collecting the leachates from drip trays on the 100th day of plant growth, the heights of plants were estimated with a measuring ribbon. All pods from each plant were counted and handpicked. Roots from every pot were carefully collected after removing the soil. Pod breadth and root length were also measured with a measuring ribbon. The grain yield (GY) of each plant was estimated on weighing balance after manually dehulling the pods. Recovered shoots and grain were dried with the help of an oven (70 °C, Memmert laboratory oven, Schwabach, Germany) after being repeatedly cleaned with distilled water till constant dry weight (DW). The roots were oven-dried after the AMF-associated parameters had been evaluated. A weighing balance was used to estimate shoot and root DW. Samples of roots, shoots, grain, and soil were kept in airtight resealable plastic bags.

### Soil and plant analysis

#### Plant Cd concentrations and nutrients in pea grain

One gram of oven-dried shoots, grain, and roots samples was separately digested using an open flask digestion apparatus containing a mixture of hydrochloric acid (HCl) and perchloric acid (HClO_4_), having a 2:1 ratio [45]. The Cd concentrations in the digests of these particular plant parts were estimated using inductively coupled plasma mass spectrometry (ICP−MS) (PerkinElmer’s NexION^®^ 2000, USA). Moreover, K, Ca, Mg, Fe, Zn, and Mn concentrations in grain digest were also measured on ICP−MS, while P concentration by the protocol of Pratt and Chapman [46].

#### Parameters of AMF

The measurements of AMF root colonization, mycorrhizal intensity (MI), frequency (MF), vesicles, arbuscules, and spore viability were carried out through various standard protocols [24]. The content of EG in 0.5 g of harvested soil was carried out as the method described earlier [42]. Similarly, TG was extracted from soil by the method described by Yuan et al. [47]. Bradford assay was carried out to estimate the contents of EG and TG in these supernatants using bovine serum albumin as the standard on a spectrophotometer [43]. The content of DG was calculated by subtracting the content of EG from TG [48].

#### Cadmium bioavailability in soil and its presence in leachates

To quantify bioavailable Cd concentration, soil samples were extracted with a DTPA solution (5 mM) [49]. Later, Cd concentrations in soil extracts and leachates were measured on ICP–MS. The immobilization index of Cd (Cd–IMDX) in soil was calculated using Eq. 1 [50].

1 $$\begin{aligned} \:\text{C}\text{d}-\text{I}\text{M}\text{D}\text{X}\:=\frac{(\text{B}\text{i}\text{o}\text{a}\text{v}\text{a}\text{i}\text{l}\text{a}\text{b}\text{l}\text{e}\:\text{C}\text{d}\:\text{i}\text{n}\:\text{C}\text{K}\:-\:\text{B}\text{i}\text{o}\text{a}\text{v}\text{a}\text{i}\text{l}\text{a}\text{b}\text{l}\text{e}\:\text{C}\text{d}\:\text{i}\text{n}\:\text{t}\text{h}\text{e}\:\text{t}\text{a}\text{r}\text{g}\text{e}\text{t}\:\text{t}\text{r}\text{e}\text{a}\text{t}\text{m}\text{e}\text{n}\text{t})}{\left(\text{B}\text{i}\text{o}\text{a}\text{v}\text{a}\text{i}\text{l}\text{a}\text{b}\text{l}\text{e}\:\text{C}\text{d}\:\text{i}\text{n}\:\text{C}\text{K}\right)}\times100\end{aligned}$$

#### Enzyme activities in soil

The activities of β-glucosidase, phosphomonoesterase, urease, catalase, and chitinase in soil were determined using spectrophotometry. For urease activity assessment, 1 g soil plus 0.5 mL urea solution and borate buffer (4 mL, having a pH of 10.0) was incubated (2 h, 37 °C). The flasks were halted (60 min) after 6 mL of potassium chloride (KCl) (1 M) was added to them. Later, water, sodium salicylate/sodium hydroxide (NaOH), and sodium dichloroisocyanurate (C_3_Cl_2_N_3_NaO_3_) were mixed with the filtrate. This mixture was placed at 25 °C for 30 min to react, and the ammonium (NH_4_^+^) concentration was detected at the optical density (690 nm) for assessing urease activity [51]. To measure β-glucosidase activity, ρ-nitrophenyl-β-D-glucopyranoside was mixed with 1 g of soil and pH buffer (pH = 9). This mixture was incubated, and later the reaction was stopped by adding Tris (0.02 mol L^−1^, having a pH of 12). Substrate cleavage released ρ-nitrophenol glucoside, discovered at 464 nm [52]. Phosphomonoesterase activity was determined after incubating soils at 37 °C while employing ρ-nitrophenyl phosphate (16 mM) as a substrate. At 400 nm, the amount of ρ-nitrophenol released during enzymatic hydrolysis was used to measure enzyme activity [53]. To measure soil chitinase activity, fresh soil (1 g), colloidal chitin suspension (1% w/w), and phosphate buffer (having a pH of 6.0) mixture was incubated (18 h, 37 °C) in a dark place. Later, the release of N-acetyl-glucosamine was measured at 585 nm [54]. For catalase activity assessment, the amount of consumed H_2_O_2_ by the soil was estimated. A formulated mixture of 25 mL H_2_O_2_ (3%) and 5 g soil was incubated (4 °C, 1 h). Then, in the 5 mL filtrate of this mixture, H_2_SO_4_ (20 mL, 0.5 M) was added. At the end, non-reactive H_2_O_2_ was measured by titrating this solution mixture against (5 mM) potassium permanganate (KMnO_4_) [55].

### Statistical analysis

Data from this experiment were interpreted with Statistix 8.1.1 (Analytical Software, Tallahassee, FL, USA) using one-way analysis of variance (ANOVA). Later, the least significant difference (LSD) test (*p* < 0.05) was performed for the determination of significant differences between treatments [56], as indicated by different lowercase letters. Data were presented in figures and tables prepared using Sigma Plot 12.5, Origin Pro 2019, and Microsoft Word 2013.

## Results

### Responses of AMF and glomalin after their crosstalk

**Fig. 1:**
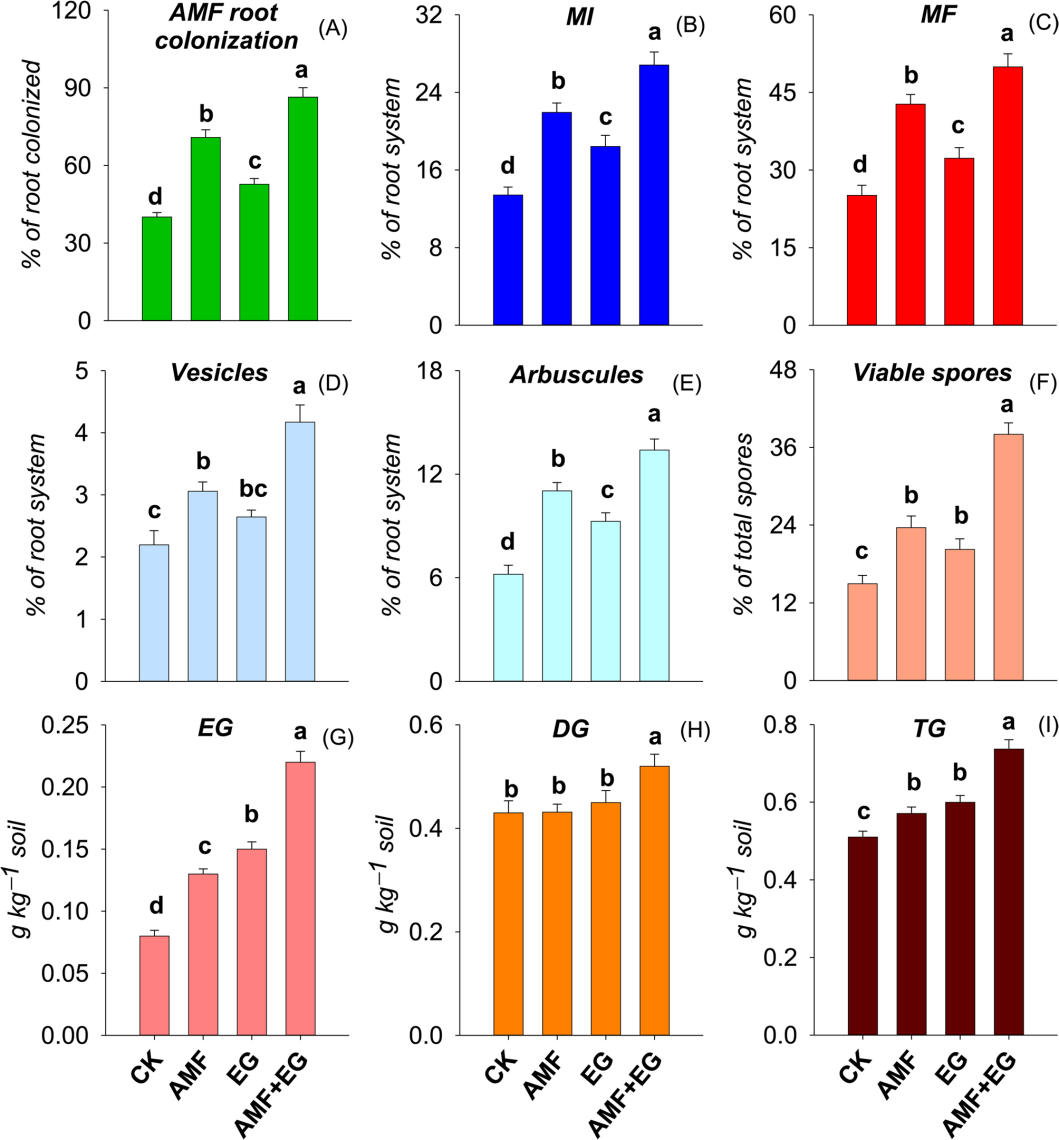
Extent of AMF root colonization (**A**), mycorrhizal intensity (MI) (**B**), mycorrhizal frequency (MF) (**C**), vesicles (**D**), arbuscules (**E**), viable spores (**F**), easily extractable glomalin (EG) (**G**), difficultly extractable glomalin (DG) (**H**) and total glomalin (TG) (**I**) in Cd-polluted soil as affected by various soil treatments. Data are displayed as mean ± standard error (*n* = 3). Different lowercase letters on bars portray significant differences between the means (*p* < 0.05)

AMF root colonization, MI and MF ranged from 40.1 to 86.4% of root colonized, 13.4−26.8% of the root system, and 25.1−50.0% of root system, respectively (Fig. 1: A − C). Whereas, data of vesicles ranged from 2.21 to 4.22% of root system, arbuscules from 6.20 to 13.4% of the root system, and viable spores from 14.9 to 38.0% of total spores, correspondingly (Fig. 1: D − F). The data about the contents of EG, DG, and TG were from 0.08 to 0.22 g kg^−1^ soil, 0.43–0.52 g kg^−1^ soil, and 0.51–0.74 g kg^−1^ soil, accordingly (Fig. 1: G − I). Compared to CK, every treatment significantly enhanced these parameters, except vesicles and DG in soil. The AMF and EG treatments did not improve the content of DG in the soil than CK. AMF root colonization, MI, MF, vesicles, arbuscules, viable spores, EG, DG, and TG were promoted up to maximum with AMF + EG treatment by 115, 101, 99, 90, 116, 155, 175, 21, and 44%, respectively, than CK.

### Soil plant available Cd, its concentrations in plants, and leachates

**Fig. 2:**
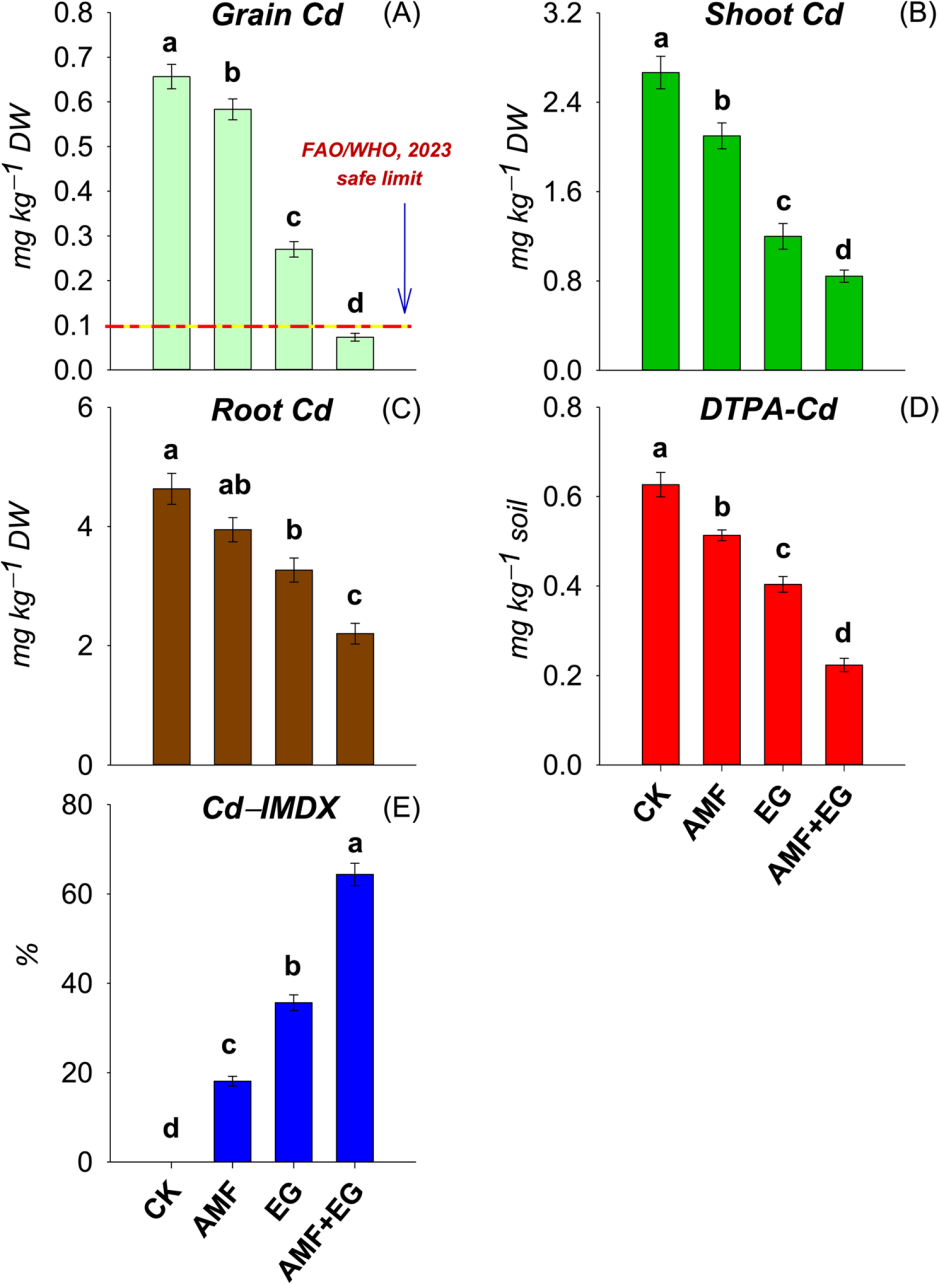
The concentrations of Cd in pea grain (Grain Cd) (**A**), shoots (Shoot Cd) (**B**), and roots (Root Cd) (**C**), while labile Cd (DTPA-Cd) (**D**) and Cd immobilization index (Cd-IMDX) (**E**) in Cd-polluted soil as affected by various soil treatments. Data are displayed as mean ± standard error (*n* = 3). Different lowercase letters on bars portray significant differences between the means (*p* < 0.05)

Cd concentrations in grain, shoots, and roots were from 0.07 to 0.66, 0.84−2.67, and 2.20−4.63 mg kg^−1^ DW in all treatments (Fig. 2). Bioavailable Cd concentration in soil was from 0.22 to 0.63 mg kg^−1^ soil, whereas, the increasing order of Cd−IMDX was AMF (18%) < EG (36%) < AMF + EG (64%) (Fig. 2). Applied soil treatments significantly reduced Cd bioavailability in soil and its concentrations in grain and shoots, than CK. AMF did not affect Cd concentration in roots, compared to CK. The AMF + EG treatment depicted the lowest Cd concentrations in grain, shoots, roots, and post-harvest soil by 89, 68, 53, and 64%, respectively, than CK. Fortunately, the Cd concentration in grain was below the FAO/WHO (2023) limit (0.1 mg kg^−1^ DW) regarding legume grain safety for human consumption in AMF + EG treatment.

**Fig. 3:**
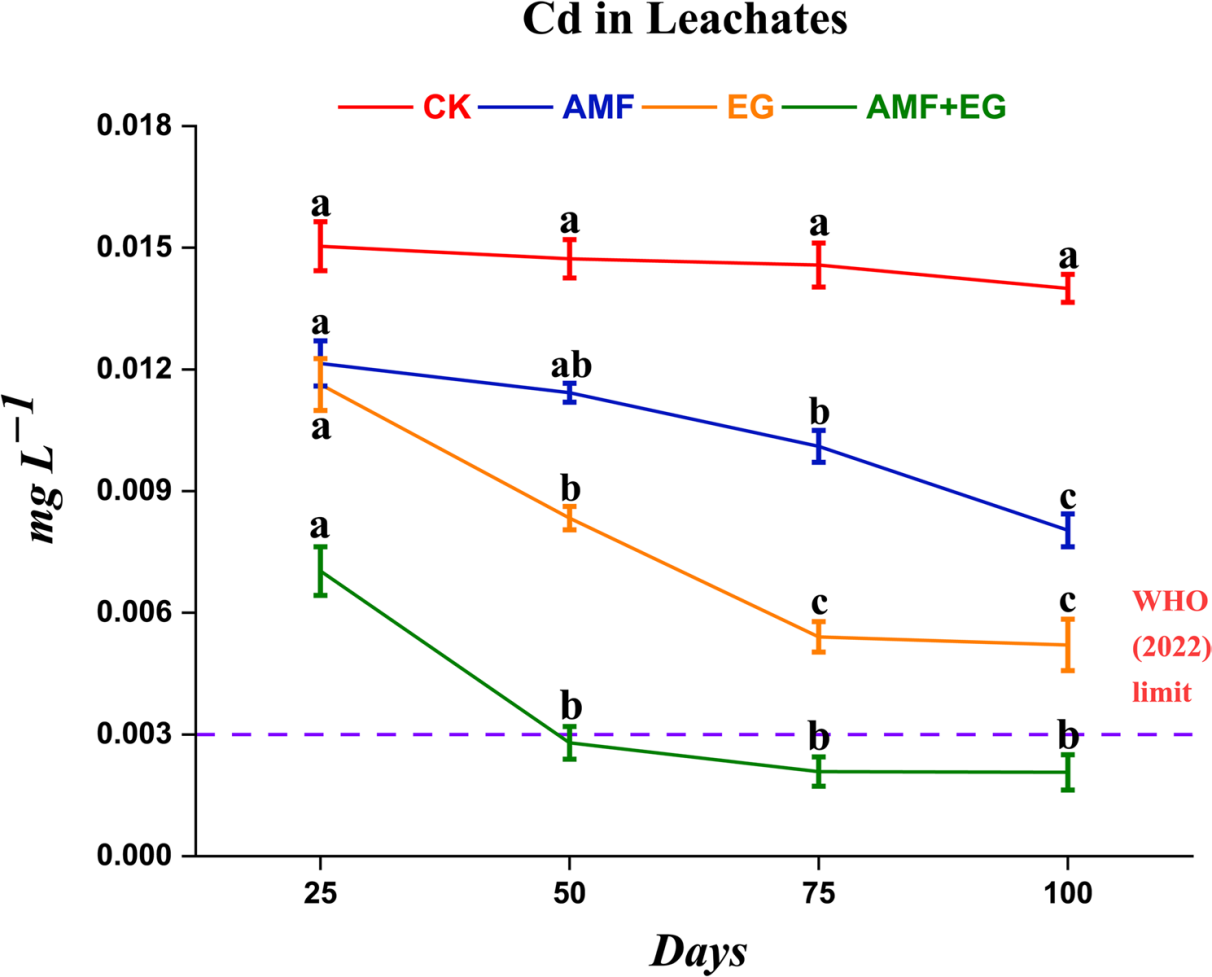
Line graph depicting time-related variations in the concentrations of Cd in leachate samples obtained from Cd-polluted soil on 25 (1st sampling), 50 (2nd sampling), 75 (3rd sampling), and 100 (4th sampling) days of the experiment as affected by various soil treatments. Every line indicates a discrete treatment. Each point in every line is the mean of three replicates with standard error and harbors different letters showing significant (*p* < 0.05) differences. Herewith, the dashed line shows the permissible limit of Cd by WHO (2022) for drinking water

The Cd concentrations in the leachates was from 0.002 to 0.015 mg L^−1^ (Fig. 3). Cd concentration in CK treatment remained unchanged at each sampling time. In AMF treatment, the concentrations of Cd at both 1st and 2nd sampling times remained virtually unchanged, while significant decreases were noted thereafter. Substantial Cd reductions were observed from the 1st sampling point to the 3rd one, but remained stable till the 4th sampling in the EG treatment. Interestingly, measured concentrations of Cd significantly declined from 1st to 2nd sampling time with AMF + EG treatment, followed by non-significant changes. This treatment ultimately kept Cd concentrations below the permissible limit (0.003 mg L^−1^) for drinking water (WHO, 2022) from 2nd to 4th sampling points. At each sampling point, the decreasing order of Cd concentrations in the leachates was CK > AMF > EG > AMF + EG.

### Plant growth, yield, and grain nutrients

**Fig. 4:**
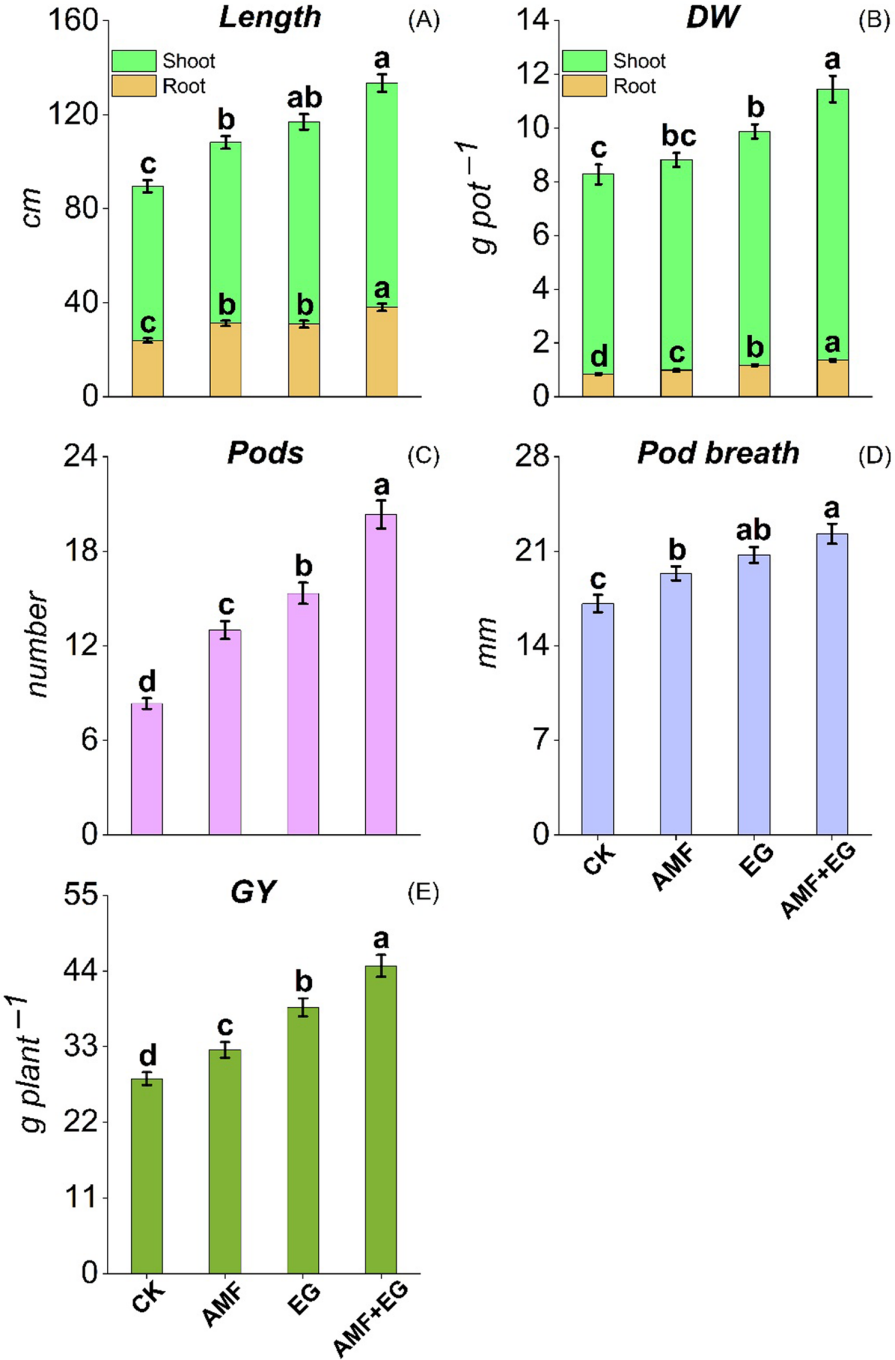
Length of shoot and root (**A**), dry weight (DW) of shoot and root (**B**), number of pods (**C**), pod breadth (**D**), and GY (grain yield) (**E**) of pea plants cultivated on Cd-polluted soil with various soil treatments. Data are displayed as mean ± standard error (*n* = 3). Different lowercase letters on bars portray significant differences between the means (*p* < 0.05)

The lengths of root, shoot, and pod breadth were 23.9–38.0 cm, 65.6–95.4 cm, and 17.1–22.3 mm, respectively. Root DW and shoot DW data varied from 0.85 to 1.36 and 7.43–10.1 g pot^−1^, respectively, while GY from 28.3 to 44.7 g plant^−1^. The number of pods remained in the range from 8.33 to 20.3 pot^−1^ (Fig. 4). Except for shoot DW in AMF treatment, all other agronomic parameters were significantly improved with each treatment than CK. Whereas, compared to CK, shoot DW was only enhanced with EG and AMF + EG treatments. The AMF + EG caused the most remarkable improvements in length of root, shoot DW, root DW, pod numbers, and GY by 59, 36, 60, 144, and 58%, correspondingly, than CK. The maximum increments of up to 31 and 45% in shoot length, while by 21 and 30% in pod breadth, were noted with EG and AMF + EG treatments, respectively, in comparison to CK.

Phosphorus and K concentrations varied in grain from 1.07 to 1.48 and 2.25–2.99 g kg^−1^ DW, respectively. Whereas Ca, Mg, Fe, Zn, and Mn concentrations in grain were from 128.7−256.4, 278.5−384.0, 37.3−62.2, 18.7−34.0, and 12.5−19.5 mg kg^−1^ DW, correspondingly (Table 1). Each treatment significantly boosted P, K, Ca, Fe, and Zn concentrations in grain than CK, except Mg and Mn in the case of AMF. The AMF + EG treatment, being the most effective, rose P, K, Ca, Mg, Fe, Zn, and Mn concentrations in grain by 38, 33, 99, 38, 67, 81 and 55%, correspondingly, compared to CK.

**Table 1:** Concentrations of various nutrients in the grain of peas cultivated on Cd-polluted soil mixed with different amendments. In each column, every value is the mean of three replicates with standard error (SE) and harbors different letters showing significant (*p* < 0.05) differences between treatments

Treatments	P	K	Ca	Mg	Fe	Zn	Mn
	*(g kg*^*−1*^ *DW)*		*(mg kg*^*−1*^ *DW)*				
CK	1.07 ± 0.04**c**	2.25 ± 0.08**c**	128.7 ± 8.6**d**	278.5 ± 12.9**c**	37.3 ± 1.76**d**	18.7 ± 0.90**c**	12.5 ± 0.52**c**
AMF	1.27 ± 0.05**b**	2.63 ± 0.09**b**	194.6 ± 9.23**c**	316.8 ± 14.2**bc**	45.9 ± 2.14**c**	29.5 ± 1.41**b**	14.7 ± 0.86**bc**
EG	1.29 ± 0.05**b**	2.67 ± 0.09**b**	227.8 ± 5.36**b**	325.1 ± 13.2**b**	53.9 ± 1.99**b**	29.2 ± 1.11**b**	16.5 ± 0.68**b**
AMF + EG	1.48 ± 0.06**a**	2.99 ± 0.10**a**	256.4 ± 10.7**a**	384.0 ± 15.9**a**	62.2 ± 3.08**a**	34.0 ± 1.51**a**	19.5 ± 0.93**a**
***LSD***_***0.05***_	0.17	0.30	28.3	46.2	7.50	4.11	2.50

### Activities of enzymes in soil

**Fig. 5:**
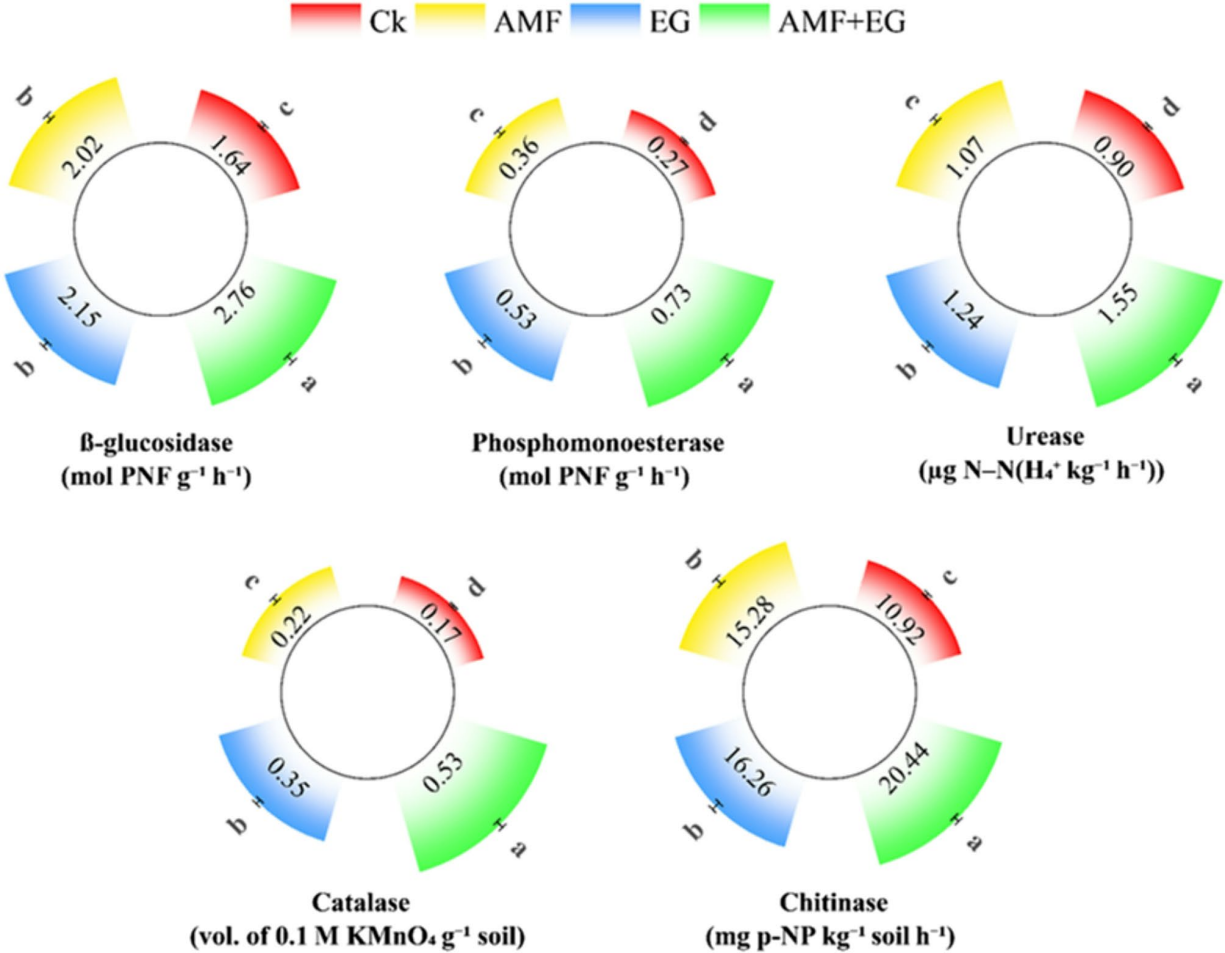
β-glucosidase, phosphomonoesterase, urease, catalase, and chitinase activities in Cd-polluted soil with various soil treatments. Data are displayed as mean ± standard error (*n* = 3). Different lowercase letters on bars portray significant differences between the means (*p* < 0.05)

The measured activities of β-glucosidase, urease, catalase, chitinase and phosphomonoesterase ranged from 1.64 to 2.76 mol PNF g^−1^ h^−1^, 0.90–1.55 µg N−N(H_4_^+^ kg^−1^ h^−1^), 0.17 − 0.53 Vol. of 0.1 M KMnO_4_ g^−1^ soil, 10.92− 20.44 mg p-NP kg^−1^ soil h^−1^, and 0.27–0.73 mol PNF g^−1^ h^−1^, respectively (Fig. 5). Significant improvements were seen in the activity of each enzyme with every treatment, than CK. The highest activities of β-glucosidase, urease, catalase, chitinase, and phosphomonoesterase by 68, 72, 206, 87, and 172%, respectively, were noted in AMF + EG treatment compared to CK.

## Discussion

### Responses of AMF and glomalin after their crosstalk

Soil Cd pollution reduces mycorrhizal root colonization, arbuscules, vesicles, viable spores, and glomalin production [24]. AMF inoculation improved MI, arbuscules [57], and vesicles [30] in the root system. In Cd-polluted soil, AMF augmented root colonization, EG and TG contents [36], and spore numbers [18]. Under orange cultivation, applying EG in soil raised soil EG content [58], while AMF root colonization with foliar applied EG [34]. The establishment of AMF-plant symbiosis (Fig. 1: A − F) and glomalin production (Fig. 1: G − I) in CK treatment was due to soil native metal-resistant AMF species. Such species never die in metal-stressed soils, produce glomalin, and establish plant symbiosis [59]. Compared to CK, AMF inoculation enhanced root colonization (Fig. 1: A − F) through (1) establishing symbiosis, (2) competing for nutrients and niches, and (3) releasing fungistatic [27]. After root colonization and hyphal proliferation have occurred, an upsurge in arbuscules and vesicle formation occurs for better nutrient acquisition and C storage [60]. AMF inoculation also increased EG and TG contents in soil than CK. AMF obtained C from plants and produced EG, which accumulated in the soil as a glomalin pool [61]. In EG treatment, exogenous application of EG to the soil enhanced AMF-plant symbiosis (Fig. 1: A − F), compared to CK. Since the exogenously applied EG was rich in essential nutrients and C-bearing compounds, which the AMF utilized, it supported the proliferation and perpetuation of the AMF in the soil [34, 58, 62]. Interestingly, the application of EG in the soil reinforced the AMF-plant symbiosis in several other ways. While acting like a glue, glomalin enhanced soil aggregation [57, 63], which augmented water retention in soil, improved soil OM content by minimizing its decomposition and reduced soil erosion [64]. Such improved soil properties, i.e., high OM content, enhanced aggregate stability, resistance against erosion, and improved water retention, in turn, served as an excellent setting for AMF colonization [28, 65, 66]. Apart from this, Cd was stabilized with externally applied EG, which ultimately reduced Cd toxicity to AMF, thereby improving the symbiosis between AMF and plants [35, 63]. Adding EG to soil raised the soil EG content [58]. Compared to CK, each treatment raised EG content, which increased soil TG content (Fig. 1: G, I), referring to increased soil-glomalin pool for C and nutrient storage [28, 66]. The content of DG was the highest with AMF + EG treatment, but did not change in AMF and EG treatments (Fig. 1: H). The turnover of EG into DG is a slow process mediated by intensive microbial activities [29, 31], depicting the highest AMF activities in AMF + EG treatment. In AMF + EG treatment, a greater number of AMF species (native AMF spp. + inoculated spp.) and a high soil EG pool (produced by native AMF spp. + externally applied) crosstalk synergistically through several mechanisms, as described earlier in this section, resulting in the strongest AMF-plant symbiosis and soil glomalin content (EG, DG and TG) (Fig. 1).

### Soil plant available Cd, its concentrations in plants, and leachates

Soil polluted with Cd escalates Cd migration in plants [60] and groundwater [18]. Contrarily, AMF reduced Cd transportation in soil and leachates [19] while its uptake in sorghum [36]. Adding glomalin efficiently adsorbed Cd in a solution [33]. At various sites, glomalin immobilized Cd up to 6.65% of total soil Cd content [67] and reduced its leaching [18]. With AMF + EG, Cd concentration in grain (0.07 mg kg^−1^ DW) was below the permissible limit of FAO/WHO [68] for legume grain safety for human consumption (Fig. 2). Whereas, this treatment also brought Cd concentrations in leachates (0.002 mg L^−1^) below permissible limit of WHO [69] for safe drinking water (Fig. 3). Moreover, the lowest Cd concentrations in roots and shoots, bioavailable Cd in soil and the highest Cd−IMDX were also noted with AMF + EG (Fig. 2). After applying AMF + EG in soil, crosstalk between AMF and EG operated synergistically and reduced Cd mobility in soil, plants, and leachates. Herewith, AMF reduced Cd mobility in soil, leachates, and plant parts because AMF sequestered Cd in spores, arbuscules, vesicles, mycelia, and hyphal cytosol [25]. AMF released EPS and EG in soil and raised PCs synthesis in plant roots, which efficiently sequestered Cd in soil and roots [24, 35]. Furthermore, a significant rise in the production of glomalin by AMF in HMs-polluted soils is reported, for which AMF utilize several resources (primarily N and C). Herein, glomalin performs protective functions for AMF against HM stress, allowing AMF to thrive in such soils [23]. Apart from this, the entry of Cd into the plant roots was restricted by AMF through physical obstruction, intracellular chelation, and alteration of the cell plasma membrane permeability [35]. Aside from AMF, the EG efficiently immobilized Cd in soil and reduced its mobility in plants and leachates by sequestering Cd via forming a clay-polyvalent Cd-OM complex and adsorbing directly on active sites [32]. Fourier Transform Infrared (FTIR) spectroscopy has already confirmed that glomalin contains functional groups such as C-H, -NH_2_, -OH, -COOH, -CO-NH, -C–O, N–H, and C=O, which efficiently immobilized Cd [33, 47, 70]. Moreover, it has already been well known that glomalin contains fluorescent substances of tyrosine-like protein, tryptophan-like protein, fulvic acid-like, humic acid-like, and soluble microbial byproduct-like, as assessed by fluorescence spectrophotometry [70]. These compounds also contributed to Cd immobilization [47]. Deprotonated sulfhydryl ligand of glomalin immobilized Cd through surface complexation and electrostatic attraction [32]. Initially, HMs saturate glomalin pores (meso and macro) and later react with internal active sites [47].

### Plant growth, yield, and grain nutrients

Plant growth and nutritional worth are reduced in Cd-polluted soil [11]. AMF inoculation in Cd-polluted soil increased sorghum height and shoot DW [36], while nutrients in pigeon pea plants [21]. Foliar-applied EG enhanced orange fruit weight, size, and nutrients [34]. In AMF + EG treatment, synergistic crosstalk between AMF and EG has occurred, which enhanced plant growth and yield (Fig. 4) and grain nutritional worth (Table 1) through several mechanisms. AMF fetched water and nutrients, crucially P, from the distal soil zones and delivered to plants [59]. Plants also got more nutrients as AMF reduced nitrification [71]. Moreover, AMF augmented plant growth through (1) raising auxin and cytokinins production and (2) regulating proline, which improved signaling, radical scavenging, protein stabilizing, and nutrient retention [35]. Interestingly, EG contained nutrients, especially C, Mg, Fe, K, Si, and humic acids, which promoted photosynthesis, C fixation, and nutrient availability to plants for better grain quality [62, 71]. Improved plant traits with glomalin are also because it improved soil (1) permeability and (2) water retention by creating a hydrophobic layer [29, 72, 73]. EG supported plant growth and yield by (1) enhancing indole-3-acetic acid (IAA) production in roots via upregulating IAA synthesis genes [62], (2) improving sucrose production, and (3) stabilizing photosystem II (PSII) complex and thylakoid membranes [71, 72]. AMF + EG reduced Cd phytotoxicity by immobilizing Cd in soil, which improved plant growth and yield [35, 67].

### Activities of enzymes in soil

Cd toxicity reduces microbial survival, their enzyme secretion and nutrient cycling capacities in soil, which retard plant growth [60]. Urease, catalase and β-glucosidase activities were enhanced in Cd-polluted soil with AMF [24]. Under variable Cd concentrations, AMF significantly improved β-glucosidase in plant rhizosphere [60]. In orchard soil, EG application improved acidic, neutral, and alkaline phosphatase activities [48]. Positive correlations between soil enzyme activities and glomalin were reported in soil [66]. In AMF + EG treatment, synergistic crosstalk between AMF and EG has occurred in soil, which collectively raised activities of soil enzymes (Fig. 5). AMF directly release urease, catalase, phosphomonoesterase, and β-glucosidase in the soil medium [59]. Moreover, enzyme secretion by microbes was further accelerated as plants allocate 4–20% C to AMF in the rhizosphere, which microbes consume [66]. AMF provided nutrients, organic substrates, and niches to microbes, enhancing their growth and capacity to secrete soil enzymes [74, 75]. AMF secreted EG and stimulated the roots to provide labile C and exudates. Labile C and these compounds accelerated soil microbial catabolic functions, enhancing soil enzyme secretion [26]. Besides, glomalin is an excellent microbial decomposition substrate that improved soil enzyme secretion by enhancing microbial catabolic functions [30]. EG, being rich in N, P, and C, acted as a nutritional source for microbes, enhancing enzyme secretion involved in the cycling of these nutrients [29, 66]. Improved soil conditions, i.e., better aeration, water retention, and soil aggregation caused by glomalin, also supported microbial activities and their enzyme secretion capacities [59, 73]. Moreover, the EG and AMF-mediated reduction in Cd toxicity to soil microbes improved their potential to secrete enzymes [25, 32].

## Conclusions

Despite manufacturing cheap earthenware (utensils and other products) and offering employment to local inhabitants, the pottery industry pollutes the soils and groundwater with Cd. This soil and groundwater pollution with Cd compromises ecological and human health. In this pot experiment, synergistic crosstalk between AMF and EG strengthened AMF-plant symbiosis and EG secretion. This synergism between AMF and EG reduced Cd bioavailability in soil and its concentrations in pea grain and leachates below permissible limits. Plant yield, grain nutritional status, and soil enzymatic activities were also the highest with AMF + EG, confirming its potential to enhance soil health and food quality. Experimental findings revealed that minute quantities of AMF inoculum and EG were highly effective in reducing the migration of Cd from the soil to plants and leachates. Such findings offer insight into a novel strategy to mitigate Cd-associated environmental and human health risks at a low cost.

### Future directions

Due to the ease of harvesting EG and the commercial availability of AMF inoculum, the efficacy of AMF + EG should be tested on a field scale. Furthermore, commercial harvesting of EG and testing the long-term effectiveness of this technique can further reduce the cost of remediating Cd-polluted soils. Future research is needed to explore the synergistic crosstalk between AMF and EG at the chemical and molecular levels.

## Data Availability

Data are provided within this manuscript.

## Abbreviations

AMF: Arbuscular mycorrhizal fungi
Cd–IMDX: Immobilization index of Cd
CEC: Cation exchange capacity
DG: Difficultly extractable glomalin
DTPA: Diethylenetriaminepentaacetic acid
EC: Electrical conductivity
EG: Easily extractable glomalin
EPS: Extracellular polymeric substances
FTIR: Fourier Transform Infrared Spectroscopy
GPx: Glutathione peroxidase
IAA: Indole-3-acetic acid
ICP−MS: Inductively coupled plasma mass spectrometry
MDA: Malondialdehyde
MF: Mycorrhizal frequency
MI: Mycorrhizal intensity
PCs: Phytochelatins
PSII: Photosystem II
SOC: Soil organic carbon
SOD: Superoxide dismutase
TG: Total glomalin
WHC: Water holding capacity
